# *Sonic hedgehog*-expressing cells in the developing limb measure time by an intrinsic cell cycle clock

**DOI:** 10.1038/ncomms5230

**Published:** 2014-07-08

**Authors:** Kavitha Chinnaiya, Cheryll Tickle, Matthew Towers

**Affiliations:** 1 MRC Centre for Developmental and Biomedical Genetics, Department of Biomedical Sciences, University of Sheffield, Western Bank, Sheffield S10 2TN, UK; 2 Department of Biology and Biochemistry, University of Bath, Claverton Down Road, Bath BA2 7AY, UK

## Abstract

How time is measured is an enduring issue in developmental biology. Classical models of somitogenesis and limb development implicated intrinsic cell cycle clocks, but their existence remains controversial. Here we show that an intrinsic cell cycle clock in polarizing region cells of the chick limb bud times the duration of *Sonic hedgehog* (*Shh*) expression, which encodes the morphogen specifying digit pattern across the antero-posterior axis (thumb to little finger). Timing by this clock starts when polarizing region cells fall out of range of retinoic acid signalling. We found that timing of *Shh* transcription by the cell cycle clock can be reset, thus revealing an embryonic form of self-renewal. In contrast, antero-posterior positional values cannot be reset, suggesting that this may be an important constraint on digit regeneration. Our findings provide the first evidence for an intrinsic cell cycle timer controlling duration and patterning activity of a major embryonic signalling centre.

## Abstract (web summary)

During vertebrate limb patterning the morphogen Sonic Hedgehog (Shh) is produced by cells of the polarizing region (ZPA). Here, the authors show, using chick embryo grafting experiments, that the duration of Shh expression by ZPA cells is defined by a cell cycle clock that is started and can also be reset by changes in retinoic acid signalling.

## Graphical abstract

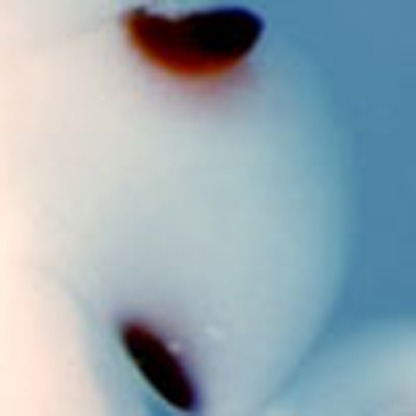

Timing is suggested as a key mechanism for specifying positional values in somitogenesis^1^,^2^,^3^ and limb development^4^. The long-standing progress zone model for patterning the proximo-distal axis (humerus-digits) of the limb is based on timing by an intrinsic cell cycle clock that runs in response to extrinsic mitogenic signals from the overlying apical ectodermal ridge^4^,^5^. In this model, the first cells displaced from the progress zone acquire proximal positional values (that is, humerus), while those displaced later more distal positional values (that is, digits). Flank-derived signals, including retinoic acid, have recently been suggested to specify proximal structures^6^,^7^,^8^, and while it is known that retinoic acid contributes to inducing *Sonic hedgehog* (*Shh*) expression^9^, it is debated whether it is required for limb patterning or just involved in limb initiation^10^,^11^. It still remains unclear whether distal positional values are specified by time^6^,^7^,^8^,^12^,^13^,^14^.

Timing is also proposed to be involved in specifying antero-posterior (thumb-little finger) positional values in the limb. The positional values for the three digits (1, 2 and 3—previous nomenclature before 2011 was 2, 3 and 4; reviewed in ref. 15) of the chick wing are specified by a paracrine morphogen gradient^16^, now known to be Shh produced by the polarizing region^17^. However, there is good evidence that the time that cells are exposed to autocrine Shh signalling specifies the positional values for the posterior digits 4 and 5 in the mouse limb that arise from the polarizing region itself^18^. To ensure a sufficient duration of Shh signalling for digit specification, and to also maintain the apical ectodermal ridge to permit outgrowth, it was suggested that loss of extrinsic signals terminates *Shh* expression at the appropriate time^19^. In this model, growth of the limb bud leads to Shh signalling being unable to maintain *Gremlin* expression (encoding a BMP-antagonist), and this, in turn, allows de-repressed BMPs to suppress FGFs in the apical ectodermal ridge that maintain *Shh* transcription. However, while *Gremlin* over-expression and application of FGF can maintain high-level *Shh* expression in the chick wing-polarizing region, the total duration of *Shh* expression is not extended for longer than usual.

Instead, we previously presented evidence for an intrinsic clock in chick wing bud-polarizing region cells related to the cell cycle from experiments in which we transiently inhibited cell cycle progression using the histone deacetylase inhibitor trichostatin A (TSA)^20^. Following cell cycle arrest, *Shh* expression was extended until a much later stage of wing development than usual, suggesting that duration of *Shh* transcription is timed with cell proliferation. We also previously showed that digit IV of the chick leg arises from the polarizing region and that progenitor cells first transit through digits I, II and III fates every 4 h (ref. 21). Therefore, an intrinsic cell cycle clock could provide a mechanism that underlies the precise promotion of these positional values in response to the duration of autocrine Shh signalling^21^. In addition, this clock could ensure the correct duration of Shh signalling to maintain the apical ectodermal ridge and thus limb bud outgrowth.

In order to test whether an intrinsic timer or the extrinsic signalling environment terminates *Shh* expression, we grafted polarizing region cells into earlier and later stage chick limb buds. We show that an intrinsic cell cycle clock in the polarizing region of the chick limb does indeed control the duration of *Shh* transcription and that timing by this clock is started and can also be reset by changes in retinoic acid signalling from the flank of the embryo.

## Results

### The polarizing region has an intrinsic timer

We carried out experiments to test further the link between cell proliferation and duration of *Shh* expression in the polarizing region of the chick wing bud using the specific mitotic inhibitor colchicine. It is possible that in our previous study^20^ the effects of TSA were due to its properties as a histone deacetylase inhibitor rather than a cell cycle inhibitor. As we previously found with TSA application, implanting colchicine-soaked beads into HH19/20 wing buds resulted in *Shh* expression being prolonged and also loss of anterior digits (Supplementary Fig. 1). We also previously showed that Shh could promote cell proliferation because inhibition of Shh signalling with cyclopamine treatment increases the proportion of cells in G1 phase in the posterior part of the wing bud^20^. To examine whether Shh promotes cell cycle progression specifically in the polarizing region we applied cyclopamine at HH20 and analysed the DNA content of polarizing region cells at 6 and 20 h by flow cytometry (Supplementary Figs 2 and 3 and Supplementary Tables 1 and 2). In both cases, cyclopamine treatment increased the proportion of cells in G1, consistent with Shh having a role in promoting cell cycle progression in the polarizing region. These data, taken together, suggest a reciprocal interaction between *Shh* expression/signalling and cell proliferation controls the duration of the polarizing region. A similar reciprocal interaction between Shh and apoptosis has previously been shown to regulate the size of the chick wing-polarizing region^22^.

**Figure 1:**
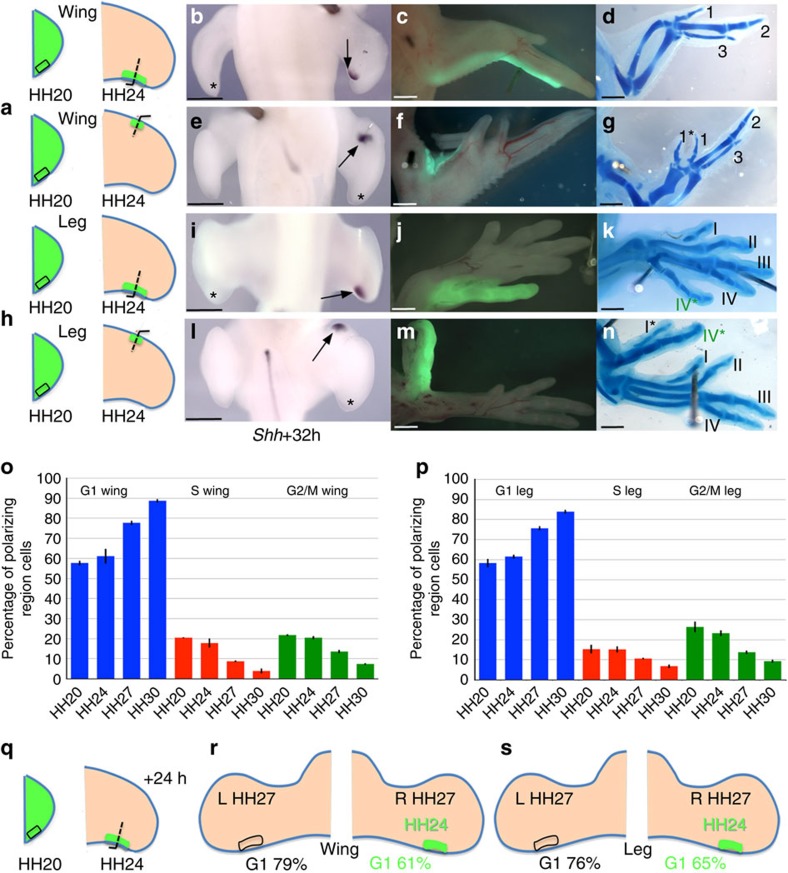
The chick limb-polarizing region has an intrinsic cell cycle clock. (**a**–**n**) HH20 wing- (**a**) and leg-polarizing regions (**h**) grafted with pins to posterior and anterior of HH24 wing- and leg-polarizing regions express *Shh* after 32 h (arrows—**b**,**e**,**i**,**l**, *n*=8/8), endogenous *Shh* undetectable (asterisks—**b**,**e**,**i**,**l**). Wings with posterior grafts normal (**c**,**d**, *n*=2/2), wings with anterior grafts have an additional digit 1* (**f**,**g**, *n*=2/2), legs with posterior leg grafts gave rise to digit IV (**j**,**k**, *n*=3/3) and anterior leg grafts to digit IV* and induce a digit I* (**m**,**n**, *n*=2/2). (**o**–**p**) Cell cycle parameters of wing- (**o**) and leg-polarizing regions (**p**)—bars indicate s.e.m. (**q**–**s**) HH20-polarizing regions grafted in place of HH24 wing- and leg-polarizing regions (**q**). After 24 h, 61 and 65% of cells in grafted wing (*n*=10, **r**) and leg (*n*=11, **s**) polarizing regions, respectively in G1 compared with 79 and 76% of cells in equal numbers of contralateral limb bud-polarizing regions. In both experiments there is a significant difference in G1 numbers between host and donor polarizing regions (Pearson’s *χ*^2^ test—*P*<0.05) consistent with graft behaving intrinsically. All scale bars, 1 mm.

*Shh* is expressed at high levels in the chick wing-polarizing region for 36–42 h (from HH18–25), then at low levels for around another 18 h (until HH27/28; Supplementary Fig. 4, see methods for staging). Previously, we reported that only the first 12 h of *Shh* expression is required for specifying the antero-posterior positional values for the digits in the chick wing (16 h in the chick leg)^21^, suggesting that *Shh* continues to be expressed in the polarizing region in order to maintain the apical ectodermal ridge. We investigated whether duration of *Shh* expression is an intrinsic property by grafting green fluorescent protein (GFP)-expressing polarizing regions from the posterior margin of HH20 wing and leg buds either in place of host polarizing regions or to anterior margins of HH24 buds (Fig. 1a,h). After 32 h, the grafted cells continued to express *Shh* robustly—consistent with their age and not host age (Fig. 1b,e,i,l). Wings with posterior grafts had a normal digit pattern (Fig. 1c,d), but consistent with the timed programme of *Shh* expression, posterior and anterior leg polarizing region grafts gave rise to a digit IV (Fig. 1j,k,m,n). However, although anterior grafts in both wing and leg buds expressed high-level *Shh*, they induced only an additional digit 1 (Fig. 1f,g,m,n), because by HH24 host mesenchyme has lost the competence to form a complete digit pattern^23^. These data show that cells of the chick limb-polarizing region have an intrinsic programme of *Shh* expression and specification of digit positional values.

### The polarizing region timer is linked with the cell cycle

Intrinsic timers have been linked to the cell cycle^1^,^2^,^3^,^4^,^5^,^24^,^25^, therefore we performed flow cytometry with cells from wing- and leg-polarizing regions. We found that between HH20 and HH30 there is a significant increase in G1 phase cells and decrease in S phase cells, indicating progressively less proliferation and/or longer cell cycling times (Fig. 1o,p, Supplementary Table 3). To test whether these cell cycle parameters are linked with timing of *Shh* expression, HH20 wing and leg polarizing regions were again grafted in place of host HH24-polarizing regions (Fig. 1q). After 24 h, 61 and 65% of cells in grafted wing and leg polarizing regions were in G1 phase, respectively, consistent with their age (HH24) and not host age (HH27; Fig. 1r,s, Supplementary Table 4; also see Fig. 1o,p). Note that G1 phase values of left and right buds deviate by <1% and are not significantly different (Supplementary Fig. 2 and Supplementary Table 1). Thus polarizing region cells have an intrinsic cell cycle clock that could time the duration of *Shh* expression.

### The polarizing region timer can be reset

**Figure 2:**
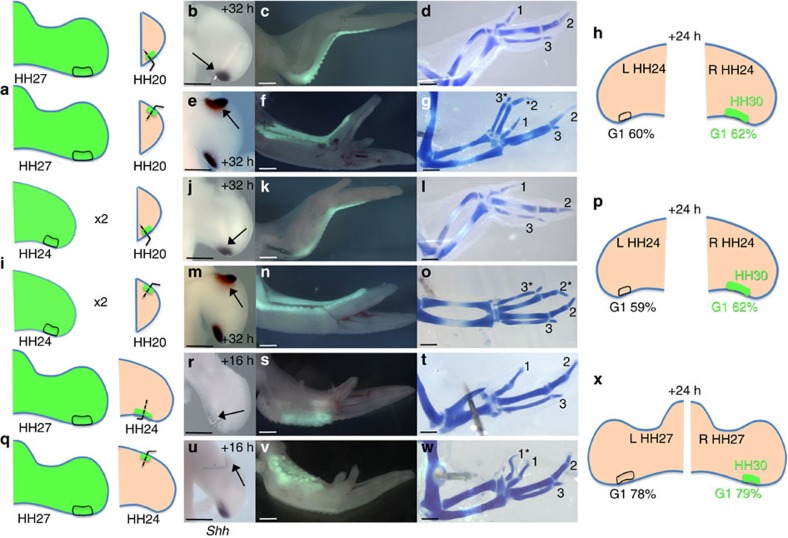
The chick wing-polarizing region intrinsic timer can be reset. (**a**–**h**) HH27 wing-polarizing region grafted to posterior and anterior of HH20 wings (**a**) express *Shh* after 32 h (arrows, **b**,**e**, *n*=11/13). Wings with posterior grafts normal (**c**,**d**, *n*=2/3—one lost digit 3), wings with anterior graft duplicated* (**f**,**g**, *n*=10/12). After 24 h, 62% of cells in G1 phase in grafted polarizing regions (*n*=10) compared with 60% in contralateral wings (**h**, *n*=10). (**i**–**p**) HH24 wing-polarizing region serially grafted (twice) to posterior and anterior of HH20 wings (**i**) express *Shh* after 32 h (arrows, **j**,**m**, *n*=3/3). Wings with posterior grafts normal (**k**,**l**, *n*=2/2), wings with anterior grafts duplicated* (**n**,**o**, *n*=2/3). After 24 h, 62% of cells in G1 phase in grafted polarizing regions (*n*=10) compared with 59% in contralateral wings (**p**, *n*=10). (**q**–**x**) HH27 wing-polarizing region grafted to posterior or anterior of HH24 wings (**q**), *Shh* not expressed after 16 h (arrows, **r**,**u**, *n*=15/15). Wings with posterior graft normal (**s**,**t**, *n*=3/3), wings with anterior graft have duplicated digit 1* (**v**,**w**, *n*=2/4—two not duplicated). After 24 h, 79% of cells in polarizing regions (*n*=12) in G1 phase compared with 78% in contralateral wing bud-polarizing regions (**x**, *n=12*). In **h**,**p** and **x** there is a significant difference in G1 numbers between host values and expected values for the stage of the donor polarizing region (Pearson’s *χ*^2^ test—*P*<0.05) consistent with cell cycle parameters of the graft being reset close to host levels (see also Supplementary Table 3). Scale bars, 500 μm (**b**,**e**,**j**,**m**), 750 μm (**r**,**u**), 1 mm (**c**,**d**,**f**,**g**,**k**,**l**,**n**,**o**,**s**,**t**,**v**,**w**).

Having shown that the polarizing region has an intrinsic cell cycle clock, we tested whether it could be reset by grafting late wing polarizing regions (HH27—expressing low-levels of *Shh* in a small domain (Supplementary Fig. 4) to early wing buds (HH20, Fig. 2a). At 32 h, *Shh* expression had increased dramatically in the grafts and was equivalent in size to the host-polarizing region (Fig. 2b,e). Posterior grafts resulted in wings with normal patterns (Fig. 2c,d), while anterior grafts induced duplicate patterns (Fig. 2f,g).

A substantial increase in the level/extent of *Shh* expression in grafts of HH27-polarizing regions to HH20 buds was already apparent by 12 h (Supplementary Figs 4 and 5), although the domain is still somewhat smaller than the endogenous domain of expression at the posterior margin of the host wing bud. This suggests that there are polarizing region cells present at stage HH27 that do not express detectable levels of *Shh* but can re-express it again under appropriate conditions. However, it is possible that increased proliferation at very early stages in the grafts could also contribute to re-establishing *Shh* expression. By 24 h, 62% of grafted cells were in G1 phase close to the 60% value of the host age (Fig. 2h and Supplementary Table 4), rather than the 89% value expected for the donor (Fig. 1o and Supplementary Table 3), showing that re-setting of the cell cycle parameters had occurred. Grafts from limbs older than HH27 made to HH20 buds failed to express *Shh* or reset cell cycle parameters (Supplementary Fig. 6 and Supplementary Table 4).

Remarkably, resetting can occur more than once as shown when HH24 grafts were made twice to HH20 buds (Fig. 2i). Thus, after a total of 86 h, *Shh* was still expressed at high levels in grafted polarizing regions (Fig. 2j,m); this is much longer than the normal duration of high-level *Shh* expression (~36 h; Supplementary Fig. 4). Buds with posterior grafts were normal (Fig. 2k, l), while anterior grafts induced duplicated digit patterns (Fig. 2n,o). Cell cycle parameters were also reset by 24 h (Fig. 2p and Supplementary Table 4), raising the possibility that polarizing region cells have the potential to proliferate and self-renew indefinitely.

Resetting of *Shh* expression timing only occurs in grafts made to early wing buds. When HH27 wing-polarizing regions were grafted to HH24 wing buds (Fig. 2q), *Shh* expression was undetectable in the grafts after 16 h (Fig. 2r,u). Posterior grafts resulted in normal wings (Fig. 2s,t), but anterior grafts still induced a digit 1 (Fig. 2v,w) presumably because of residual Shh in the graft. Unexpectedly, however, after 24 h, G1 phase values appeared to have been reset and were almost identical in left (78%) and right (79%) buds (Fig. 2x and Supplementary Table 4). This suggests that polarizing region cells not expressing *Shh* have a similar cell cycle profile to the endogenous polarizing region that expresses low-levels of *Shh* at this late stage (Supplementary Fig. 4). These data, taken together, show that timing of *Shh* expression and the cell cycle clock can be reset in the chick wing-polarizing region by extrinsic signals, although re-setting of *Shh* expression requires an earlier environment.

### Positional values cannot be reset in the polarizing region

**Figure 3:**
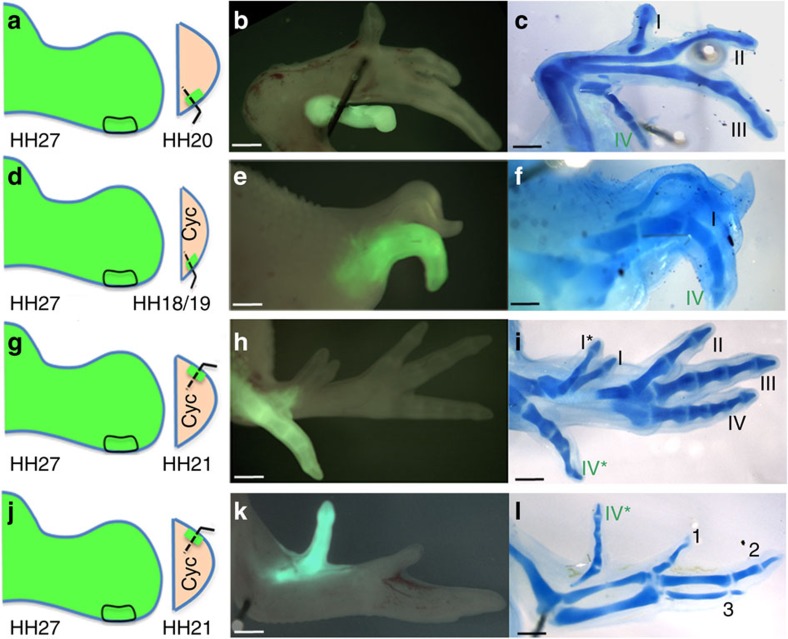
Positional value of chick leg-polarizing region cells is irreversibly specified. (**a**–**c**) HH27 leg-polarizing region grafted to posterior of HH20 host-polarizing region (**a**) give rise to digit IV (**b**,**c**, *n*=3/4; one case no digit from graft). (**d**–**f**) HH27 leg-polarizing region grafted to posterior of HH18/19 leg-polarizing region treated with cyclopamine (**d**) gives rise to digit IV (**e**,**f**, *n*=3/4 one case limb truncated). (**g**–**i**) HH27 leg-polarizing region grafted to anterior margin of HH21 leg treated with cyclopamine (**g**) give rise to digit IV *, a duplicate digit I* from host (**h**,**i**, *n*=2/3, one case no duplicated host digits, note extra digit IV* (**i**) is out of plane with the other digits and appears posterior although it is an anterior digit. (**j**–**l**) HH27 leg-polarizing regions grafted to anterior margins of HH21 wing treated with cyclopamine (**j**) give rise to digit IV* (**k**,**l** *n*=2/2). All scale bars, 1 mm.

To understand whether the positional values of the digits are intrinsically determined we turned to the chick leg in which the polarizing region cells are promoted every 4 h through digit identities (I, II and III) before giving rise to digit IV (ref. 21). As in wings, *Shh* expression and cell cycle parameters can also be reset in leg-polarizing region grafts (HH27 grafts to HH20 leg buds; Fig. 3a, Supplementary Fig. 7 and Supplementary Table 4). In addition, leg-polarizing region grafts gave rise to digit IV (Fig. 3b,c), just as grafts of HH20 leg polarizing region to HH20 buds^21^.

To test whether specification of the leg digit IV had been reset in the early environment, or whether its fate irreversibly specified, we grafted HH27 leg-polarizing regions either posteriorly to HH18/19 leg, or anteriorly to HH21 leg and wing buds (Fig. 3d,g,j) and simultaneously treated with cyclopamine to curtail Shh signalling. If resetting occurs and Shh signalling is attenuated, the leg-polarizing region should give rise to a more anterior digit, because digit progenitor cells transit through digits I, II and III fates in response to Shh signalling^21^. However, the grafts still gave rise to digit IV, despite attenuated Shh signalling, as shown by failure to specify digit III in adjacent host tissue (Fig. 3e,f,h,i,k,l—note application of cyclopamine after HH21 does not affect host digit pattern^21^). Therefore, these data show that *Shh* expression and signalling are dispensable for the late-stage polarizing region to differentiate into a digit IV when grafted into the early bud. Additionally, that the anterior to posterior sequence of positional values that specify leg digit IV are irreversible and cannot be reset by any extrinsic signals present in either early wing or leg buds.

### Retinoic acid depletion starts the polarizing region timer

**Figure 4:**
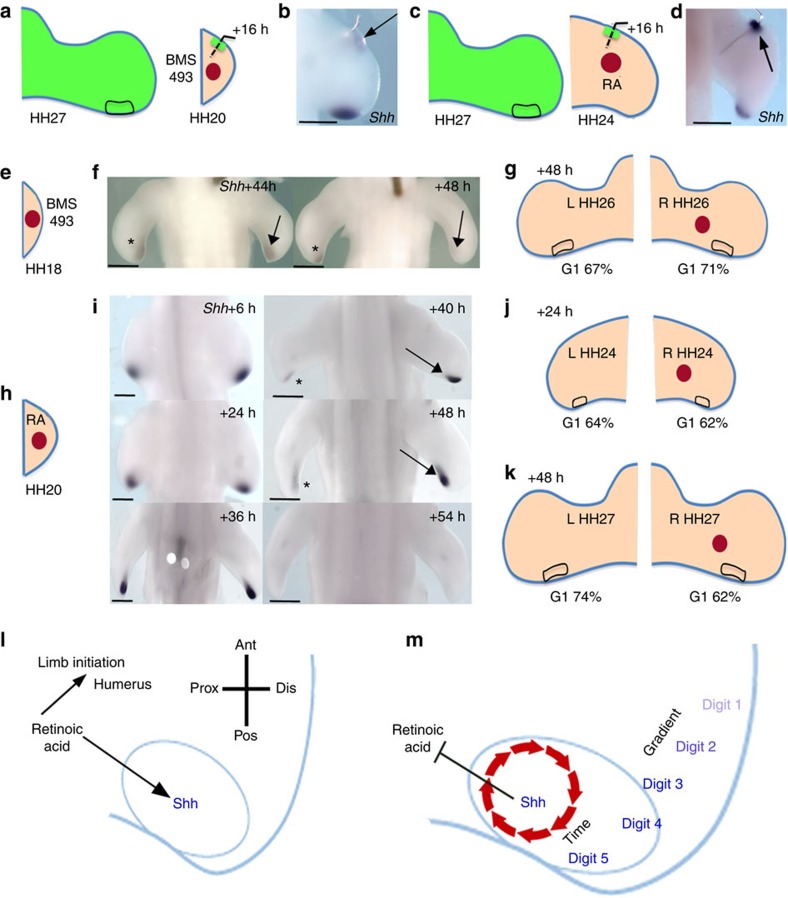
Retinoic acid influences the chick wing-polarizing region timer. (**a**,**b**) HH27 wing-polarizing region grafted to anterior margin of BMS-493 bead-treated HH20 wings (**a**) have reduced (or undetectable) *Shh* expression after 16 h (arrow, **b**, *n*=10/16—compare with untreated wings; Fig. 2e and Supplementary Fig. 5). (**c**,**d**) HH27 wing-polarizing region grafted to anterior margin of retinoic acid (RA) bead-treated HH24 wings (**c**) express *Shh* after 16 h (arrow, **d**, *n*=3/3—compare with untreated wings; Fig. 2u). (**e**–**g**) HH18 wing buds treated with BMS-493 terminate *Shh* expression 4 h earlier (arrows, **f**, *n*=5/7) than in untreated wings (asterisks, **f**). After 48 h, 71% of polarizing region cells in treated wing buds (*n*=11) compared with 67% in untreated buds (*n*=11, **g**). (**h**–**k**) HH20 wings treated with retinoic acid beads (**h**) express *Shh* at high levels 10–12 h longer (arrows, **i**) than untreated wings (asterisks, **i**, *n*>5 for each time-point). After 24 h (**j**) 62% of polarizing region cells in G1 phase in treated wing buds (*n*=12) compared with 64% in untreated buds (*n*=12) and after 48 h (**k**) 62% of polarizing region cells in treated wing buds (*n*=12) compared with 74% in untreated buds (*n*=12). (**l**–**m**) *Shh*-expressing limb bud cells measure time by an intrinsic cell cycle clock. In early buds (**l**) retinoic acid promotes limb initiation^10^,^11^ and is suggested to specify proximal structures (that is, humerus)^7^,^8^ and be required for *Shh* transcription in the polarizing region (oval, **l**)^9^,^17^. Shh then contributes to clearing retinoic acid from the early bud^28^ and our data suggest that this starts an intrinsic cell cycle clock (red circle, **m**) that times *Shh* duration. Graded paracrine Shh signalling specifies digits 1, 2 and 3 (ref. 21) while autocrine Shh signalling specifies digit 4 in the chick leg^21^ (also digit 5 in the mouse limb^18^, **m**). The cell cycle clock enables polarizing region cells to measure time of *Shh* expression and thus irreversibly acquire antero-posterior positional values. For **g**,**j** and **k**, Pearson’s *χ*^2^ tests reveal a significant difference (*P*-value<0.05) in G1 phase cell numbers between treated and untreated buds. Scale bars, 500 μm (**b**,**i**; left panels), 750 μm (**d**,**f**,**i**; right panels).

A candidate factor for the resetting of *Shh* expression is retinoic acid that is present proximally in early wing buds^26^,^27^. When we grafted HH27 wing-polarizing regions anteriorly to HH20 wing buds treated 4 h earlier with the retinoic acid receptor antagonist BMS-493 (Fig. 4a) *Shh* expression levels in the grafts were reduced at 16 h (Fig. 4b, compare with Fig. 2e and Supplementary Fig. 5), although normal posterior expression of *Shh* was unaffected (Fig. 4b). In addition, when HH27 wing-polarizing regions were grafted anteriorly to HH24 wing buds treated 4 h earlier with retinoic acid (Fig. 4c), *Shh* was expressed robustly in the grafts at 16 h (Fig. 4d, compare with Fig. 2u). However, application of retinoic acid to HH25 buds failed to prolong endogenous *Shh* transcription, suggesting that resetting is only possible in host buds that are HH24 or younger (Supplementary Fig. 8). Although it is difficult to rule out other possibilities, resetting of *Shh* could share parallels with initiation of *Shh* expression and depend on other factors only present in the early bud that act together with retinoic acid. These findings indicate that retinoic acid is the endogenous proximal factor that resets *Shh* expression and controls the duration of *Shh* expression.

To test this further we treated HH18 wing buds at the onset of *Shh* expression with BMS-493 (Fig. 4e) to inhibit retinoic acid signalling during the narrow time-window before it becomes depleted and timing of *Shh* expression starts. This resulted in the duration of *Shh* expression being shortened by ~4 h (Fig. 4f), the polarizing region having a higher proportion of G1 phase cells at 48 h (71%) compared with control buds (67%; Fig. 4g and Supplementary Table 4) and is consistent with the intrinsic polarizing region timer being started earlier than normal. To examine conversely whether retinoic acid treatment can delay the onset of the polarizing region timer, we treated HH20 wing buds with retinoic acid to maintain signalling during outgrowth (Fig. 4h). Retinoic acid treatment did not affect *Shh* expression in early buds but strikingly high-level *Shh* expression was extended for ~10–12 h in treated buds compared with control buds (Fig. 4i). In addition, in buds in which retinoic acid beads were implanted at HH20 and replaced with fresh beads 24 h later at HH24, polarizing region cells maintained a G1 phase value of 62%, typical of early wings for at least 48 h (embryos then at HH27); the G1 phase value of polarizing region cells in the contralateral HH27 wing bud is 74% (Fig. 4j,k, Supplementary Table 4; also see Fig. 1o).

## Discussion

This study has given new insights into a little understood but important area of developmental biology—how time is measured during pattern specification. Our data suggest the duration of *Shh* expression in polarizing region cells is determined by an intrinsic cell cycle clock and this clock could provide a mechanism for providing the polarizing region cells with positional information based on the time they are exposed to autocrine Shh signalling and also could ensure the appropriate timing of bud outgrowth (Fig. 4l,m). The clock is reminiscent of the intrinsic cell cycle clock previously described in cultured oligodendrocytes^25^. However, in showing that a similar clock operates *in vivo*, and also controls the duration of a key embryonic signalling centre, our findings are unprecedented. We have also shown that following the onset of *Shh* expression in the early bud, the intrinsic cell cycle clock starts once polarizing region cells fall out of range of retinoic acid signalling by the flank and this times the remaining duration of *Shh* expression (Fig. 4l,m). In this respect, there is another parallel with the oligodendrocyte intrinsic clock that is set by extrinsic hydrophobic signals such as thyroid hormone or retinoic acid^25^. One possibility is that retinoic acid has a general role in controlling the activity of embryonic timers, including those that time cell differentiation as in cultured oligodendrocytes and those like the one operating in the polarizing region that we have described here that time the duration of cell–cell signalling. We also have shown that retinoic acid treatment can stop the clock in the polarizing region cells and allow it to be reset. The programmes of *Shh* expression and cell cycle can be then be recapitulated, possibly indefinitely. In this respect, *Shh*-expressing cells are behaving like self-renewing stem cell populations. In contrast, our experiments show that positional values in the polarizing cells are not reset and this could be one of the factors that act to constrain regenerative ability as the chick limb bud develops.

## Methods

### Chick husbandry and polarizing region grafts

Hamilton Hamburger stages (HH) are used throughout. HH18 occurs at ~3.25 days of incubation, HH20-day 3.5, HH23-day 4, HH24-day 4.5, HH26-day 5, HH27-day 5.5, HH28/29-day 6, HH29/30-day 6.5 and HH30-day 7. Fertilized Brown Leghorn chicken eggs were incubated at 38 °C for 3 days (HH17) and windowed using blunt forceps to make a small opening in the side of the shell, which was then covered with clear tape. The eggs were re-incubated and left until the desired stage of development (see above). GFP-expressing embryos were dissected in DMEM (Gibco) and limb bud-polarizing regions removed using sharpened tungsten needles, grafted to equivalently sized holes cut into the anterior or posterior margins of the limb buds of host embryos and held in place with 25 μm platinum pins (Goodfellow Metals).

### Chemical treatment

Cyclopamine (5 μl; Sigma) dissolved in control carrier (45% 2-hydroxypropyl-β-cyclodextrin in PBS; Sigma) to a concentration of 1 μg μl^−1^ was pipetted directly into the egg after removal of vitelline membranes using fine forceps. Retinoic acid (Sigma) and BMS-493 (Sigma and a gift from M. Torres) and colchicine (Sigma) were dissolved in DMSO (Sigma) to 5 μg μl^−1^ (retinoic acid and BMS-493) or 0.05μg μl^−1^ (colchicine). Formate-derivatized AG1-X2-beads (Sigma-150 μm diameter—sieved using nylon mesh to exclude smaller beads) were soaked in these compounds for 30 min, washed twice in DMEM and implanted into chick wing buds. For 48 h retinoic acid treatment, beads were replaced after 24 h with freshly prepared ones.

### Skeletal preparations

Embryos were fixed in 90% ethanol for 2 days then transferred to 0.05% alcian blue in 80% ethanol/20% acetic acid for 1 day. Embryos were then rehydrated through an ethanol series before being cleared in 1% KOH.

### Whole mount *in situ* hybridization

Embryos were removed from eggs, vitelline membranes removed using fine forceps and then fixed overnight in 4% PFA at 4 °C. Embryos were dehydrated and rehydrated through a methanol series, washed in PBS, then treated with proteinase K for 20 mins (10 μg ml^−1^). Embryos were washed twice for 5 min in PBS, fixed for 30 min in 4% PFA and then pre-hybridized at 65 °C for 2 h (50% formamide/50% 2 × SSC). Antisense mRNA probe (1 μg) for *Shh* was added to 1 ml of hybridization buffer (50% formamide/50% 2 × SSC) at 65 °C overnight. Embryos were washed in hybridization buffer, and then in maleic acid buffer (MAB) buffer, before being transferred to blocking buffer (2% blocking reagent 20% lamb serum in MAB buffer) for 2 h at room temperature. Embryos were transferred to blocking buffer containing anti-digoxigenin antibody (1/2,000; Roche) at 4 °C overnight, washed in MAB buffer and transferred to NTM buffer containing nitroblue tetrazolium chloride/5-bromo-4-chloro-3-indolyl-phosphate and *Shh* expression visualized.

### Cell cycle analyses by flow cytometry

Normal and GFP-expressing limb bud-polarizing regions were dissected in PBS under a LeicaMZ16F UV microscope using fine surgical scissors, pooled from replicate experiments (between 10 and 12), and digested into single-cell suspensions with trypsin (0.5%, Gibco) for 30 min at room temperature. Cells were washed twice in PBS, fixed in 70% ethanol overnight, washed twice in PBS and re-suspended in PBS containing 0.1% Triton X-100, 50 μg ml^−1^ of propidium iodide and 50 μg ml^−1^ of RNase A (Sigma). After incubation at room temperature for 20 min, cells were analysed for cell cycle distribution with a FACSCalibur flow cytometer and FlowJo software (Tree star Inc). Cells with a DNA content between 2N and 4N were designated as being in the G1, S, or G2/M phase and expressed as a percentage of the total number of cells present (6,000–10,000). Statistical significance of cell cycle phase values was determined by Pearson’s *χ*^2^ tests to obtain two-tailed *P*-values (significantly different being a *P*-value of <0.05).

## Author contributions

K.C. performed all flow cytometry, cyclopamine experiments and implanted BMS-493/retinoic acid beads and discussed results; C.T. edited drafts of the paper and discussed results; M.T. designed the project, performed grafts and all other experiments, first in the lab of C.T., then in his own lab and wrote the paper.

## Additional information

**How to cite this article**: Chinnaiya, K. *et al.* *Sonic hedgehog*-expressing cells in the developing limb measure time by an intrinsic cell cycle clock. *Nat. Commun.* 5:4230 doi: 10.1038/ncomms5230 (2014).

## Supplementary Material

Supplementary Information Supplementary Figures 1-8 and Supplementary Tables 1-5
