# Supplementary material for: Sonic hedgehog-expressing cells in the developing limb measure time by an intrinsic cell cycle clock
Source: Nat Commun. 2014 Jul 8;5:4230. doi: 10.1038/ncomms5230 (PMC4102111; doi:10.1038/ncomms5230)
Supplement: Supplementary Information — Supplementary Figures 1-8 and Supplementary Tables 1-5 [file ncomms5230-s1.pdf]

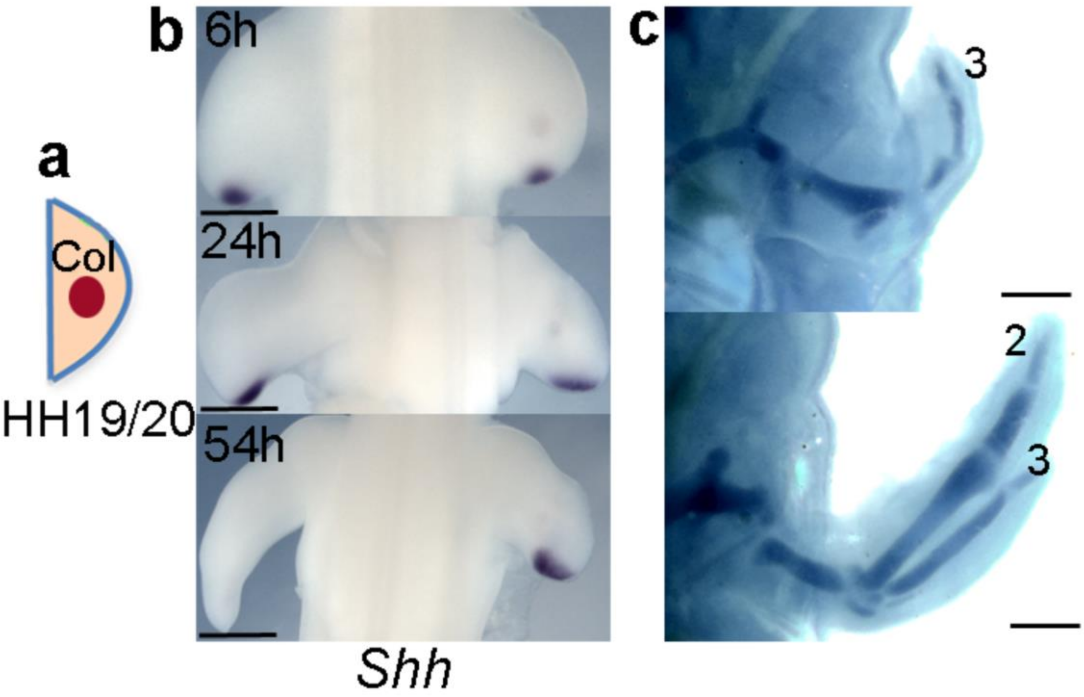

Supplementary Figure 1 - Colchicine extends *Shh* duration and causes anterior digit loss in the chick wing

Colchicine (Col)-soaked beads implanted into HH19/20 chick wing buds (**a**) extend duration of high-level *Shh* expression by around 20hrs (right buds, **b,** *n*=10/10-compare with left buds) and transient growth inhibition leads to loss of anterior digits 1 and 2 (top panel, **c**, *n*=4/6) or digit 1 only (bottom panel, **c**, *n*=2/6). Note application of the histone deacetylase inhibitor Trichostatin A (TSA) also extends *Shh* expression and causes loss of anterior digits. However, following TSA application *Shh* transcription is immediately inhibited for around 20hrs. Scale bars-**b** top panel-500μm, middle panel-750μm, bottom pattern-1mm; **c**-1mm.

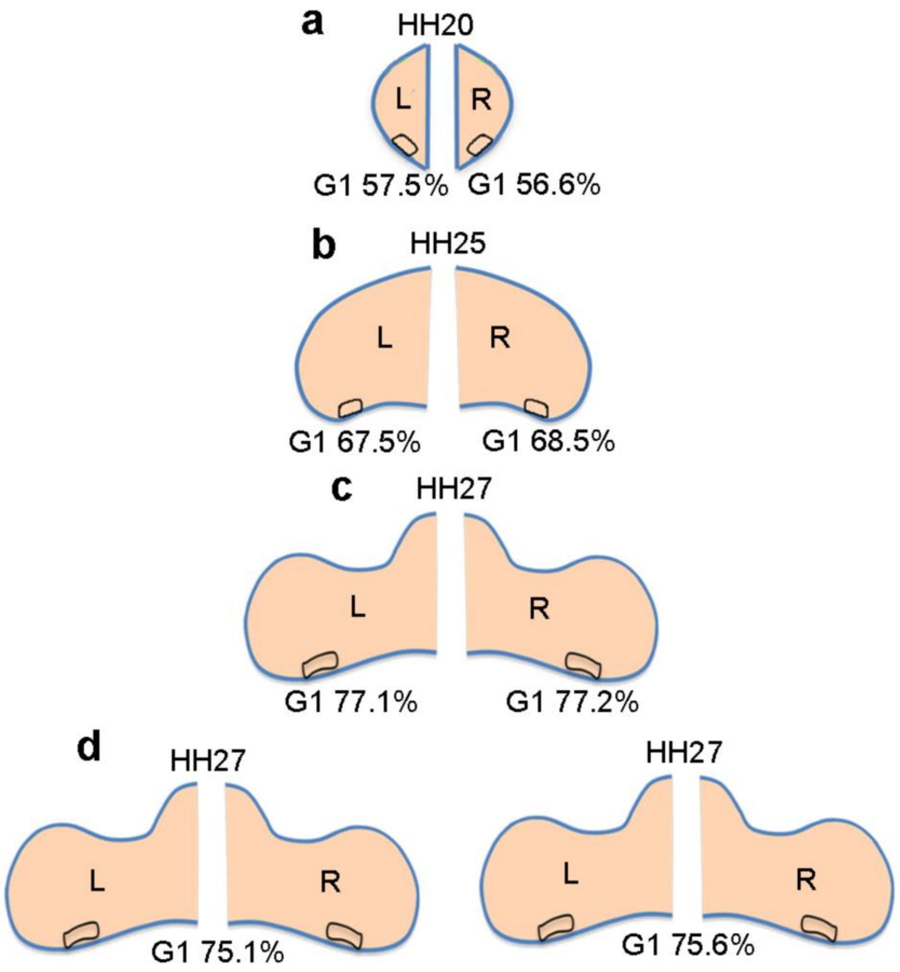

Supplementary Figure 2 – Controls for flow cytometry showing percentages of cells in G1 phase in left and right chick wing polarizing regions from the same embryos and pooled left and right polarizing regions from separate embryos

a-c compares cell cycle profiles in the polarizing regions of left (*n*=10 in all cases) and right (*n*=10 in all cases) wing buds of the same embryos. **d** compares pooled left (*n*=10) and right polarizing regions (*n*=10) from separate embryos collected at the same time. Pearson's chi-squared test on total cell numbers reveals no significant difference (p value > 0.05) in percentages of cells in G1 phase in left and right buds (**a-c**) and from separate embryos (**d**). See Supplementary Table 1.

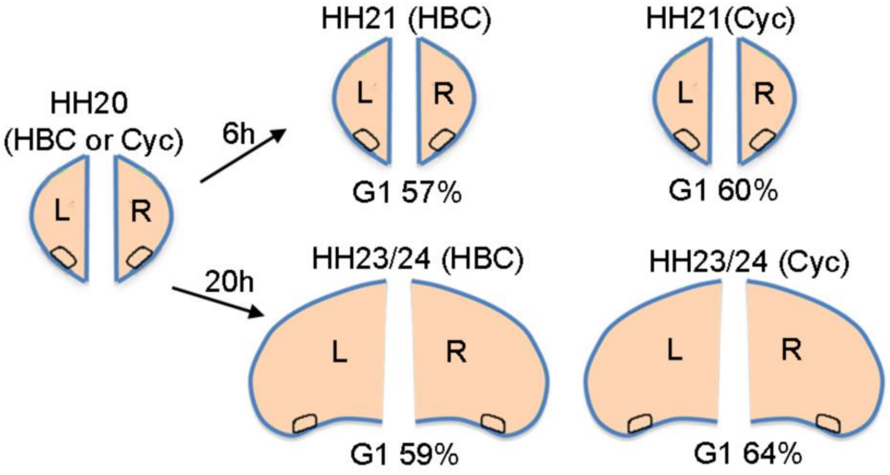

Supplementary Figure 3 - Percentages of G1 phase cell numbers in cyclopamine and HBC control treated chick wing polarizing regions

Percentages of cells in G1 phase in chick wing polarizing regions 6hrs and 20hrs following treatment at HH20 with cyclopamine (Cyc) conjugated to hydroxypropyl-β-cyclodextrin (HBC) and controls treated with HBC only (see Supplementary Table 2). Pearson's chi-squared test on total cell numbers reveals a significant difference (p value <0.05) in G1 phase percentages between polarizing regions of cyclopamine and HBC treated embryos (n=12 in all cases) showing that cyclopamine increases the number of cells in G1 phase (see also Supplementary Figure 2 and Supplementary Table 1 showing no significant difference is found when separate pools of normal polarizing regions are analysed).

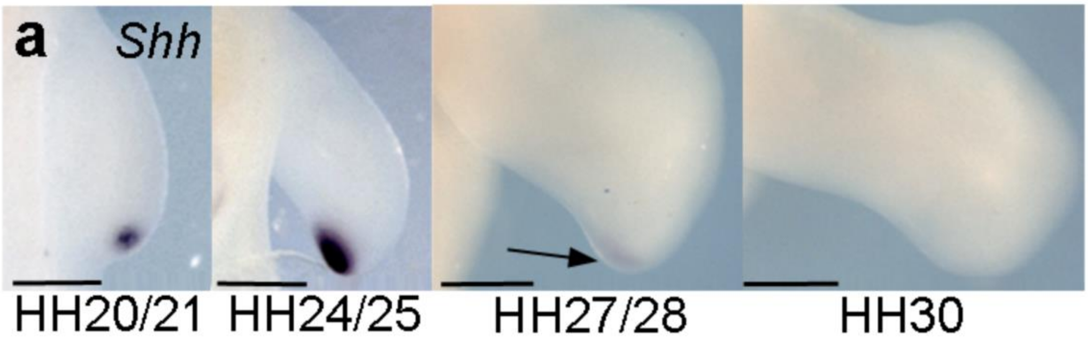

Supplementary Figure 4 – *Shh* expression in the chick wing polarizing region

Shh is expressed in the chick wing polarizing region at high-levels until HH24/25, low-levels until HH27/28 (arrow) and is undetectable by HH30. Scale bars all 500µm.

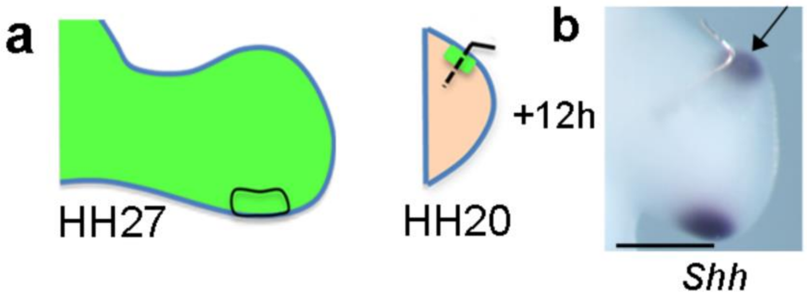

Supplementary Figure 5 - *Shh* expression in HH27 to HH20 wing polarizing region grafts after 12hrs

HH27 GFP-expressing chick wing polarizing regions grafted to the anterior margins of HH20 host polarizing regions (**a**). The domain of *Shh* expression (arrow) is substantially increased compared with the domain at the time of grafting (n=8/8, see Supplementary Fig. 4) although it is still smaller than the domain of *Shh* expression in the posterior polarizing region (**b**). Scale bar 500μm.

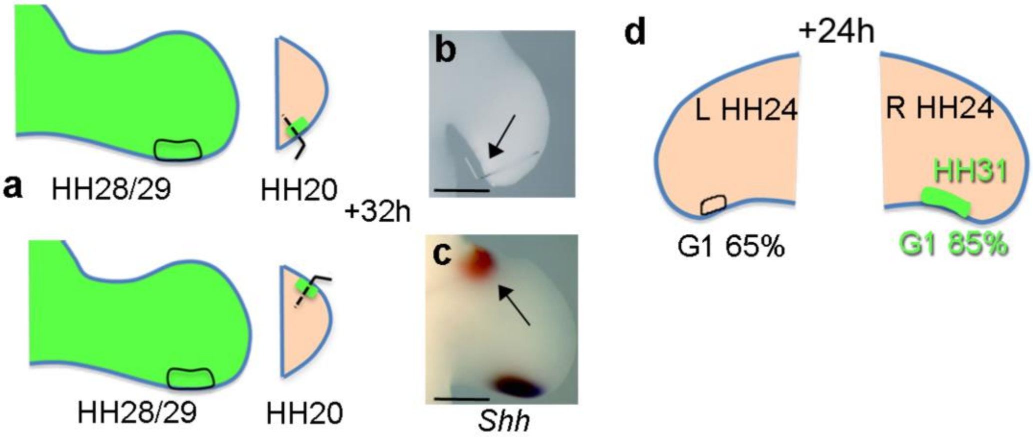

Supplementary Figure 6 - Late stage chick wing polarizing regions fail to reset *Shh* expression and cell cycle parameters

HH28/29 GFP-expressing chick wing polarizing regions grafted in place of HH20 host polarizing regions and to anterior margins of HH20 wings (**a**) do not express detectable *Shh* after 32hrs (arrows, **b**, n=5/5, note red staining in **c** is *Gfp* expression). After 24hrs, 85% of cells in G1-phase in grafted polarizing regions (n=11) compared to 65% in contralateral polarizing regions (n=11, **d**, Supplementary Table 4). Pearson's chi-squared test on total cell numbers shows a significant difference in G1 phase percentages ($p<0.05$) between host values (65%) and donor values (85%) consistent with the graft maintaining its cell cycle parameters – see Supplementary Table 3). Scale bars 500μm.

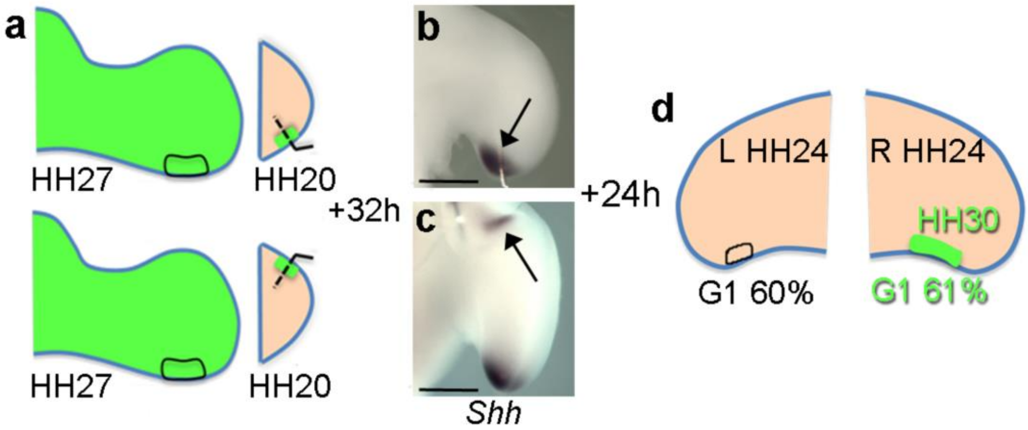

Supplementary Figure 7 - The chick leg polarizing region intrinsic timer can be reset

HH27 chick leg polarizing region grafted to posterior and anterior of HH20 leg buds (**a**) express *Shh* after 32hrs (arrows, **b**, **c**, *n*=4/6) After 24hrs, 61% of cells in G1-phase in grafted polarizing regions (*n*=10) compared to 60% in contralateral leg polarizing regions. (*n*=10, **d**). Pearson's chi-squared test on total cell numbers shows a significant difference ($p<0.05$) in G1 phase percentages between host values (60%) and expected values for the stage of the donor polarizing region (84% - Supplementary Table 4, see also Supplementary Table 3) consistent with cell cycle parameters of the graft being reset close to host levels. Scale bars 500µm.

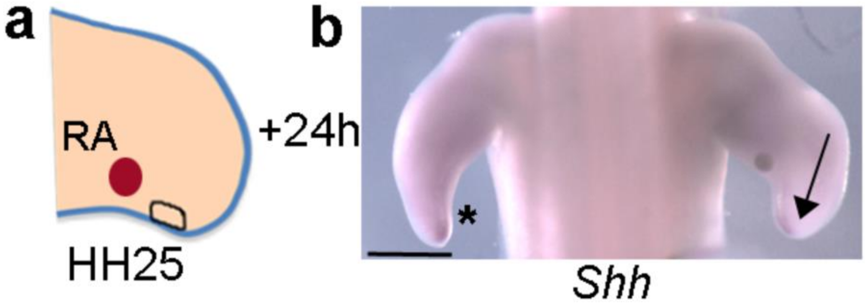

Supplementary Figure 8 - Late application of retinoic acid does not affect *Shh* expression in the chick wing

Retinoic acid (RA) applied on beads to HH25 chick wing buds (**a**) does not affect *Shh* expression after 24hrs (arrow, **b** *n*=10/10) compared with untreated buds (asterisk, **b**). Scale bar 750µm.

Stage	G1	S	G2/M	Stage	G1	S	G2/M	Figure
HH20 (R)	56.6	20.7	22.7	HH20 (L)	57.5	20.4	22.1	S2
HH25 (R)	68.5	16.4	15.1	HH25 (L)	67.5	16.8	15.7	S2
HH27 (R)	77.2	8.4	14.4	HH27 (L)	77.1	8.5	14.4	S2
HH27 (R+L)	75.6	10.1	14.3	HH27 (R+L)	75.1	10.9	14.0	S2

Supplementary Table 1 – Controls for flow cytometry showing cell cycle profiles of left and right chick wing polarizing regions from the same embryos and pooled left and right polarizing regions from separate embryos

The top three rows compare cell cycle profiles in the left (L) and right (R) wing buds of the same embryos. The bottom row compares pooled left and right polarizing regions from separate embryos harvested at the same time. In all cases between 6000 and 10000 cells were counted from 10 polarizing regions.

Cyc	G1	S	G2/M	HBC	G1	S	G2/M	Figure
6hrs HH21	60.2	17.6	22.2	6hrs HH21	56.5	21.3	22.2	S3
20hrs HH23/24	64.3	14.5	21.2	20hrs HH23/24	59.4	17.5	23.1	S3

Supplementary Table 2 - Analyses of cell cycle profiles in chick wing polarizing regions following cyclopamine treatment at HH20

HH20 embryos treated with cyclopamine (cyc) conjugated to hydroxypropyl-β-cyclodextrin (HBC) and controls treated with HBC only. Middle row shows results after 6hrs at HH21, bottom row after 20hrs at HH23/24. In each experiment 12 left and right polarizing regions were pooled and 6000 to 10000 cells were counted.

Stage	G1 wing	S wing	G2/M wing	Stage	G1 leg	S leg	G2/M leg	Figure
HH20 (3)	57.7	20.5	21.8	HH20 (3)	58.3	15.3	26.4	1o, p
HH24 (3)	61.7	17.8	20.5	HH24 (3)	61.5	15.2	23.3	1o, p
HH27 (3)	77.7	8.7	13.6	HH27 (3)	75.6	10.6	13.8	1o, p
HH30 (2)	88.7	3.9	7.4	HH30 (2)	83.9	6.8	9.3	1o, p

Supplementary Table 3 - Analyses of cell cycle profiles in chick wing and leg polarizing regions

Numbers in brackets refers to number of repeat experiments for each stage – the average values are shown. Standard Error is indicated on the graphs in Figures 1o and p. In each experiment between 6000 and 10000 cells were counted from 10 to 14 pooled polarizing regions.

Graft	Right stage	G1	S	G2/M	Left stage	G1	S	G2/M	Figure
HH20-24 Wing	HH24	61.1	20.0	18.9	HH27	79.4	8.8	11.8	1r
HH20-24 Leg	HH24	65.4	12.0	22.6	HH27	76.3	10.6	13.1	1s
HH27-20 Wing	HH30	61.6	17.4	21.0	HH24	60.1	17.7	22.2	2h
HH28/29-20 Wing	HH31	84.8	6.1	9.1	HH24	65.4	12.7	21.9	S6
HH27-20 Leg	HH30	60.9	13.7	25.4	HH24	60.1	14.4	25.5	S7
HH24-20 x2 Wing	HH30	62.4	20.3	17.3	HH24	58.9	20.8	20.3	2p
HH27-24 Wing	HH30	78.9	9.6	11.5	HH27	77.7	12.0	10.3	2x

Supplementary Table 4 - Percentages of chick limb polarizing region cells in different cell cycle phases in right and left buds of the same embryos following grafting experiments

Stage of host (left) and donor (right) tissue is shown (note stage of the right donor polarizing region shown in second column is the age it would have been if left in situ – see Supplementary Table 3 for expected values). In each experiment 10 to 12 polarizing regions were pooled and between 6000 and 10000 cells were counted.

Treatment	Stage	G1(R)	S (R)	G2/M(R)	G1 (L)	S (L)	G2/M (L)	Figure
HH18 +BMS493 48hrs	HH26	71.1	13.6	15.3	66.7	15.2	18.1	4g
HH20+RA 24hrs	HH24	61.7	13.7	24.6	64.4	12.8	22.8	4j
HH20+RA 48hrs	HH27	62.3	15.5	22.2	74.1	13.3	12.6	4k

Supplementary Table 5 - Analyses of cell cycle profiles in chick wing polarizing regions following BMS-493 or retinoic acid treatment

Right (R) wing buds treated either with BMS-493 or retinoic acid (RA), left buds (L) untreated controls. 10 to 12 polarizing regions were pooled and 6000 to 10000 cells were counted.
